# Different patterns of NF-κB and Notch1 signaling contribute to tumor-induced lymphangiogenesis of esophageal squamous cell carcinoma

**DOI:** 10.1186/1756-9966-30-85

**Published:** 2011-09-22

**Authors:** Chunhua Su, Zhenguang Chen, Honghe Luo, Yihua Su, Wangkai Liu, Lie Cai, Tao Wang, Yiyan Lei, Beilong Zhong

**Affiliations:** 1 Department of Thoracic Surgery, The First Affiliated Hospital, Sun Yat-sen University, Guangzhou (510080), Guangdong, People's Republic of China; 2 Department of Ophthalmology, The First Affiliated Hospital, Sun Yat-sen University, Guangzhou (510080), Guangdong, People's Republic of China; 3 Department of Pediatrics, The First Affiliated Hospital, Sun Yat-sen University, Guangzhou (510080), Guangdong, People's Republic of China; 4 Department of Rehabilitation, The First Affiliated Hospital, Sun Yat-sen University, Guangzhou (510080), Guangdong, People's Republic of China; 5 Center for Stem Cell Biology and Tissue Engineering, Sun Yat-sen University, Key Laboratory for Stem Cells and Tissue Engineering, Ministry of Education, Guangzhou, Guangdong 510080, China; 6 Department of Thoracic Surgery, The Fifth Affiliated Hospital, Sun Yat-sen University, Zhuhai (519000), Guangdong, People's Republic of China

**Keywords:** esophageal squamous cell carcinoma, Notch, NF-κB, angiogenesis, lymphangiogenesis

## Abstract

**Background:**

Lymph node involvement and tumor-induced lymphangiogenesis appear as the earliest features of esophageal squamous cell carcinoma (ESCC), although the molecular regulatory mechanisms involved have remained unclear. Our aim was to investigate the contribution of NF-κB and Notch1 signaling to lymph node involvement and tumor-induced lymphangiogenesis in ESCC.

**Material and methods:**

NF-κB and Notch1 expression in 60 tissue samples of ESCC were assessed by immunohistochemical staining. The correlations of NF-κB and Notch1 with lymph node involvement, lymphatic vessel density (LVD), podoplanin, and vascular endothelial growth factor-C (VEGF-C) were further evaluated to determine the association of NF-κB and Notch1 expression with tumor-induced lymphangiogenesis.

**Results:**

Chi-square tests revealed that NF-κB and Notch1 expression in ESCC tissues were significant associated with lymph node metastasis, LVD, podoplanin, and VEGF-C expression. Strong expression of NF-κB, but weak expression of Notch1, was observed in tumor tissues with lymph nodes involvement (*P *< 0.05 for both). The mean histoscores of LVD, podoplanin, and VEGF-C staining were higher in high-NF-κB-expressing tissue than in low-expressing tissue (*P *< 0.05 for each). In contrast, the mean histoscores of LVD and VEGF-C staining were lower in high-Notch1-expressing tissue than in low-expressing tissue (*P *< 0.05 for both). A multiple factors analysis of LVD and VEGF-C further demonstrated that LVD and VEGF-C status were significantly correlated with NF-κB and Notch1 expression in tumors. NF-κB and Notch1 expression were also significantly inversely correlated (*P *< 0.05).

**Conclusion:**

These results suggest that different patterns of NF-κB and Notch1 signaling contribute to lymph nodes metastasis and tumor-induced lymphangiogenesis of ESCC, and reveal that up-regulation of NF-κB is associated with down-regulation of Notch1 in tumor tissue.

## Background

Esophageal squamous cell carcinoma (ESCC) is one of the most aggressive and invasive malignancies in the world. Despite combined modality approaches, the prognosis in cases of ESCC remains extremely poor; patients exhibit a low 5-year survival rate, with the majority of cancer-related deaths resulting from metastatic spread of tumor cells [1]. Clinical observations have shown that lymph node involvement appears as one of the earliest features of ESCC [2]. Some abnormal molecular biology changes, such as tumor-induced lymphangiogenesis, are also considered to play a central role in the migration and metastatic spread of ESCC to lymph nodes. For example, high expression of vascular endothelial growth factor (VEGF)-C and the presence of newly developed lymphatic ducts was found to be the main avenue for dissemination of malignant cells to lymph nodes in ESCC [3-5]. Lymphangiogenesis is associated with neoplastic progression in the esophageal mucosa, and there is an increase in VEGF-C expression in Barrett's epithelium as it progresses through dysplasia to esophageal carcinoma [6]. Moreover, lymphangiogenesis has been shown to correlate with the depth of malignant invasion, tumor stage, lymphatic and venous invasion, and lymph node metastasis in esophageal cancer [7].

However, although several positive and negative regulators, including angiopoietins [8], neuropilin-2 [9], and COX-2 [10], are believed to contribute to the robust production of VEGF-C, the molecular regulatory mechanisms involved in tumor-induced lymphangiogenesis of ESCC have remained unclear. One potential candidate is nuclear factor-κB (NF-κB), a sequence-specific transcription factor that responds to cellular signaling pathways involved in cell survival and resistance to chemotherapy; notably, aberrant NF-κB activation has been associated with some malignancies [11-13]. Although abnormities of NF-κB signaling have been reported to play an important role in carcinogenesis by promoting tumor-induced angiogenesis and neoplastic proliferation [14], the association of NF-κB with lymphangiogenesis in ESCC is less clear. Members of the Notch family of cell surface receptors and their ligands also warrant attention based on their role in vasculogenesis and their potential to act as oncogenes in the pathogenesis of certain carcinomas. These highly conserved proteins regulate "decisions" involved in cell-fate determination, including those involved in mammalian vascular development [15]. The finding that genes of the Notch signaling cascade are robustly expressed in the vasculature suggests that Notch signaling guides endothelial cells and associated mural cells through the cell-fate decisions needed to form and maintain the vascular system [16]. Although Notch signaling anomalies are found in melanoma, non-small cell lung cancer, cervical cancer and neuroblastoma, consistent with the presumed oncogenic role of Notch signaling during tumorigenesis, the finding that Notch signaling is diminished in epithelial squamous cell carcinoma of the skin would seem to suggest that Notch might serve as a tumor suppressor. These apparently contradictory functions of Notch signaling strongly indicate that the outcome of Notch activation is dependent on malignant cellular context [17].

Given the uncertain contributions of differential NF-κB and Notch signaling to tumor-induced lymphangiogenesis of ESCC, we here assessed the expression of NF-κB and Notch1 in ESCC tissues and evaluated their association with various clinical characteristics, including sex, age, lymph node metastasis, tumor-node-metastasis (TNM) classification, and differentiation (well, moderate, or poor grade) of tumor cells in ESCC. Lymphangiogenetic characteristics and their associations with NF-κB and Notch1 signaling were also measured to determine the contribution of NF-κB and Notch signaling to tumor-induced lymphangiogenesis.

## Materials and Methods

### Patients and specimens

A total of 60 ESCC tissue samples excised from January 2004 to December 2006 were selected from the Department of Thoracic Surgery of the First Affiliated Hospital, Sun Yat-sen University. All patients were treated by esophagectomy and did not receive chemotherapy or radiotherapy before surgery. Clinical information was obtained through reviews of preoperative and perioperative medical records, or telephone or written correspondence. These cases were classified according to the Health Organization criteria (TNM system) and staged appropriately. The study has been approved by the hospital ethical committee and each subject had signed the written informed consent.

### Pathological grading

Paraffin-embedded specimens of each case were collected, and 5-mm thick tissue sections were cut and fixed onto siliconized slides. The histopathology of each sample was studied using hematoxylin and eosin (H&E) staining. The same sections were deparaffinized and rehydrated with deionized water. Samples were stained with hematoxylin for 5 min and ablated with 1% hydrochloric acid alcohol for 30 s then immersed in distilled water for 15 min. Slides were stained with 0.5% eosin for 2 min, then dehydrated, immersed in xylene for 15 min, and mounted. All specimens were evaluated with respect to histological subtype, differentiation, and tumor stage according to World Health Organization criteria. Tumor size and metastatic lymph node number and locations were obtained from pathology reports.

### Immunohistochemical staining

Immunohistochemical staining was carried out using the streptavidin-peroxidase method. Briefly, each tissue section was deparaffinized, rehydrated, and then incubated with fresh 3% hydrogen peroxide (H_2_O_2_) in methanol for 15 min. After rinsing with phosphate-buffered saline (PBS), antigen retrieval was carried out by incubating at 100°C for 15 min in 0.01 M sodium citrate buffer (pH 6.0) using a microwave oven. Next, non-specific binding was blocked by incubating with normal goat serum for 15 min at room temperature, followed by incubation at 4°C overnight with anti-NF-κB antibody (sc-8008, 1:500; Santa Cruz Biotechnology, Santa Cruz, CA, USA), anti-Notch1 antibody (sc-6014-R, 1:500; Santa Cruz Biotechnology), anti-VEGF-C antibody (18-2255, 1:100; Invitrogen, Carlsbad, CA, USA), anti-VEGFR-3 antibody (MAB3757, 1:150; Chemicon, Santa Cruz, CA, USA), and/or anti-podoplanin antibody (sc-59347, 1:100; Chemicon, Santa Cruz, CA, USA). After rinsing with PBS, slides were incubated for 10 min at room temperature with biotin-conjugated secondary antibodies, followed by incubation with a streptavidin-conjugated peroxidase working solution for 10 min. Subsequently, sections were stained for 3-5 min with 3,3'-diaminobenzidine tetrahydrochloride (DAB), counterstained with Mayer's hematoxylin, dehydrated, and mounted. Negative controls were prepared by substituting PBS for primary antibody.

### Assessment of immunohistochemical staining

Nuclear staining of NF-κB and cytoplasmic staining of Notch1 and VEGF-C were scored in this study. The intensity of NF-κB, Notch1, podoplanin, and/or VEGF-C staining was score on a scale of 0-3 as follows: 0, negative; 1, light; 2, moderate; and 3, intense. The percentage of positive tumor cells at each intensity level was presented as a ratio of the percentage of surface area covered at each intensity score to total tumor cell area. Areas that were negative were given a value of 0. We analyzed 10-12 discrete foci in each section and generated an average stain intensity and percentage of surface area covered. The final histoscore was calculated using the formula, histoscore = (1 × percentage of weakly positive tumor cells) + (2 × percentage of moderately positive tumor cells) + (3 × percentage of intensely positive tumor cells). The histoscore was determined independently by two investigators by microscopic examination (magnification, × 400). If the histoscores determined by the two investigators differed by more than 15%, a recount was taken to reach an agreement. NF-κB, Notch1, podoplanin, and VEGF-C expression were classified into high- and low-expressing groups, using the median value of their respective histoscores as a cut-off value.

### Evaluation of LVD

Immunohistochemical reactions for VEGFR-3 antigen were evaluated independently by two investigators using a microscope. The three most vascularized areas within a tumor ("hot spots") were chosen at low magnification (× 40), and vessels in a representative high-magnification (× 400; 0.152 mm^2^; 0.44-mm diameter) field in each of these three areas were counted. The high-magnification fields were then marked for subsequent image cytometric analysis. Single immunoreactive endothelial cells or endothelial cell clusters separated from other micro-lymphatic vessels were counted as individual micro-lymphatic vessels. Endothelial staining in large vessels with tunica media and nonspecific staining of non-endothelial structures were excluded in micro-lymphatic vessels counts. The mean visual micro-lymphatic vessel density of VEFGR-3 staining was calculated as the average of six counts (two hot spots and three microscopic fields). Micro-lymphatic vessel counts higher than the median micro-lymphatic vessel count were taken as high LVD, and those that were lower than the median were taken as low LVD.

### Statistical analysis

All calculations were done using the statistical software SPSS V.14.0 (Chicago, Illinois, USA). Data were shown as mean ± standard deviation. Spearman's coefficient of correlation, Chi-squared tests, and Mann-Whitney tests were used as appropriate. A multivariate model using logistic regression analysis was used to evaluate statistical associations among variables. For all tests, a two-sided *P*-value less than 0.05 was considered to be significant. Hazard ratios (HR) and their corresponding 95% confidence intervals (95% CI) were computed to provide quantitative information about the relevance of the results of the statistical analysis.

## Results

### Basic clinical information and tumor characteristics

Forty-six male and 14 female patients (mean age, 57.6 ± 10.4 years; range, 36-79 years) with ESCC treated by curative surgical resection were enrolled in the study. Of the 60 tumors, 15 were well differentiated, 27 were moderately differentiated, and 18 were poorly differentiated. Using the TNM staging system of the International Union Against Cancer (2009) [18], cases were classified as stage I (n = 9), stage II (n = 11), and stage III (n = 40). Twenty-four of 60 patients had lymph node metastasis, according to surgery and pathology reports. Patient data were analyzed after a 5-year follow-up; information was obtained in 91.7% (55 of 60) of cases. The median overall survival was 26.9 ± 2.7 months (95% CI: 21.4-31.9 months), and the mean overall survival was 38.1 ± 6.5 months (95% CI: 27.6-52.0 months). The clinical characteristics of study samples are summarized in Table 1.

**Table 1:** Association of NF-κB and Notch1 expression with clinical characteristics

Clinicopathological feature		NF-κB expression		*P-*value	Notch1 expression		*P*-value
				
		High	Low		High	Low	
**Gender**							
	Male	21	25	0.451	22	24	0.887
	Female	8	6		7	7	
Age (years)							
	≤ 60	17	23	0.201	23	17	0.058
	> 60	12	8		6	14	
Differentiation							
	Well	7	8	0.231	3	12	0.001
	Moderate	16	11		10	17	
	Poor	6	12		16	2	
TNM stages							
	I + II	8	12	0.361	10	10	0.855
	III	21	19		19	21	
Lymphatic metastasis							
	With	23	2	0.001	6	19	0.001
	Without	6	29		23	12	
LVD (VEGF-R3)							
	High	19	12	0.038	10	21	0.010
	Low	10	19		19	10	
Podoplanin							
	High	20	10	0.004	8	19	0.008
	Low	9	21		21	12	
VEGF-C expression							
	High	18	11	0.039	6	23	0.001
	Low	11	20		23	8	
Notch1 expression							
	High	8	21	0.002			
	Low	10	21				

### Association of NF-κB and Notch1 expression with clinical features of ESCC

The association of NF-κB expression with several clinicopathologic factors is shown in Table 1. NF-κB expression in tumor cells was significantly correlated with lymph node metastasis (*χ*^2 ^= 32.727, *P *= 0.001), LVD (*χ*^2 ^= 4.312, *P *= 0.038), VEGF-C expression (*χ*^2 ^= 4.241, *P *= 0.039), podoplanin expression (*χ*^2 ^= 8.076, *P *= 0.004), and Notch1 expression (*χ*^2 ^= 9.675, *P *= 0.002). Similarly, Notch1 expression in tumor cells was significantly correlated with lymph nodes metastasis (*χ*^2 ^= 10.162, *P *= 0.001), LVD (*χ*^2 ^= 6.362, *P *= 0.010), VEGF-C expression (*χ*^2 ^= 17.176, *P *= 0.001), and podoplanin expression (*χ*^2 ^= 6.877, *P *= 0.008). There were no associations of Notch1 or NF-κB with age, sex, or TNM stage of tumors.

### Association of NF-κB and Notch1 with lymph node metastasis in ESCC

**Figure 1:**
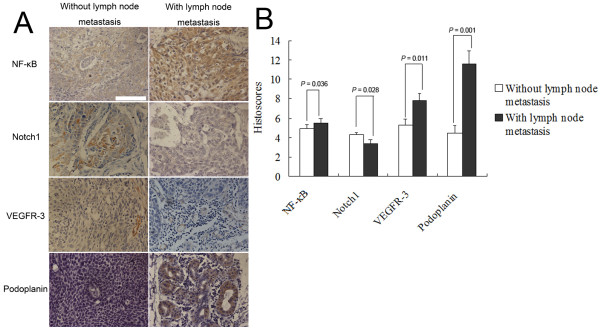
**Association of NF-κB and Notch1 expression with lymph node metastasis in ESCC**. (A) Compared with samples of ESCC without lymph node involvement, the samples of ESCC with lymph node involvement showed high levels of NF-κB expression and low levels of Notch1 expression (magnification, ×200). (B) In ESCC tissue with lymph node involvement, NF-κB staining was strong (mean histoscore, 5.55 ± 0.41) and Notch1 staining was weak (mean histoscore, 3.41 ± 0.36) compared with tissues without lymph node involvement (mean histoscores, 4.90 ± 0.43 and 4.27 ± 0.27 for NF-κB and Notch1, respectively; *P *< 0.05 for both).

In order to observe the association of NF-κB and Notch1 expression levels with lymph nodes metastasis in greater detail, we compared the histoscores of NF-κB and Notch1 expression in the context of lymph node involvement (Figure 1). Significantly, our data suggest differences in the patterns of NF-κB and Notch1 signaling with respect to lymph node metastasis status in ESCC, demonstrating strong expression of NF-κB in ESCC tissue, but weak expression of Notch1 with lymph node involvement (*P *< 0.05 for both). A multivariate analysis of lymph node involvement in ESCC (Table 2) indicated a positive association of NF-κB and VEGF-C expression with lymph node metastasis, independent of T stage, sex, age, and differentiation of tumor cells.

**Table 2:** Multivariate analysis of lymph node involvement in ESCC (logistic regression model)

Variable	*β*	HR (95% CI)	*P*
NF-κB	1.551	4.716 (1.037-21.454)	0.045
Notch1	-0.273	0.761 (0.459-1.263)	0.291
VEGF-C	0.866	2.377 (1.257-4.494)	0.008
T stage	0.117	1.125 (0.627-2.016)	0.694
Sex	-0.157	0.855 (0.160-4.566)	0.854
Age	0.030	1.030 (0.966-1.098)	0.365
Differentiation	- 0.126	0.882 (0.284-2.736)	0.828

Abbreviations: HR, hazard ratio; CI, confidence interval of the estimated HR.

### Association of NF-κB and Notch1 with tumor-induced lymphangiogenesis in ESCC

**Figure 2:**
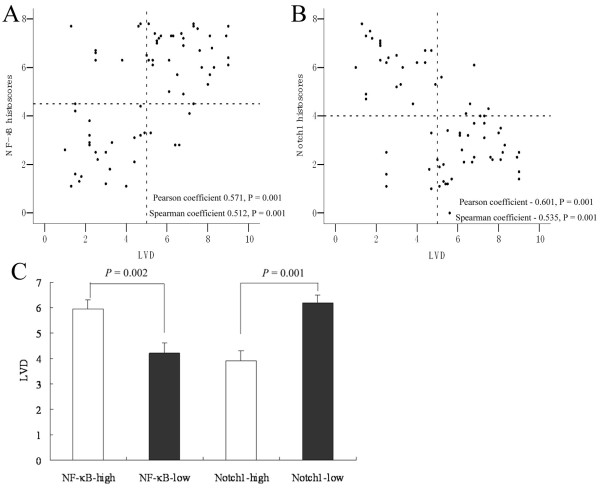
**Association of NF-κB and Notch1 expression with lymphangiogenesis in ESCC**. (A) NF-κB expression in ESCC tissue was positively correlated with LVD in tumors. (B) Notch1 expression in ESCC tissue was negatively correlated with LVD in tumors. (C) The mean histoscore of LVD expression was higher in ESCC tissue with high levels of NF-κB expression (5.95 ± 0.35) than in those with low levels of NF-κB expression (4.22 ± 0.39; *P *< 0.05). Conversely, the mean LVD histoscore (VEGFR-3 expression) was lower in ESCC tissue with high levels of Notch1 expression (3.92 ± 0.38) than in those with low levels of Notch1 expression (6.20 ± 0.31; *P *< 0.05).

**Figure 3:**
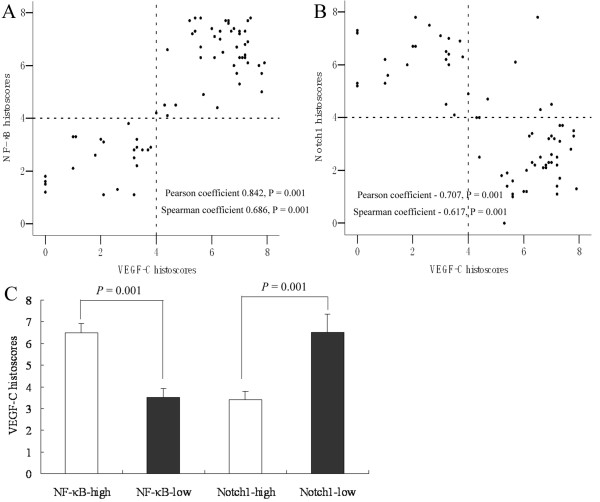
**Association of NF-κB and Notch1 expression with VEGF-C in ESCC**. (A) NF-κB expression in ESCC tissue was positively correlated with VEGF-C expression in tumors. (B) Notch1 expression in ESCC tissue was negatively correlated with VEGF-C expression in tumors. (C) The mean histoscore of VEGF-C expression was higher in ESCC tissue with high levels of NF-κB expression (6.48 ± 0.44) than in those with low levels of NF-κB expression (3.53 ± 0.39; *P *< 0.05). Conversely, the mean histoscore of VEGF-C expression was lower in ESCC tissue with high levels of Notch1 expression (3.41 ± 0.37) than in those with low levels of Notch1 expression (6.51 ± 0.84; *P *< 0.05).

The average histoscore of LVD (VEGF-R3) distribution, an important lymphangiogenetic factor, was 5.06 ± 0.28 in all ESCC samples in our study. LVD histoscores were higher (5.95 ± 0.35) in NF-κB-high patients and lower (4.23 ± 0.39) in NF-κB-low patients (Figure 2). Conversely, lower rates of LVD were observed in Notch1-high patients (3.92 ± 0.38), whereas higher rates were found in Notch1-low patients (6.20 ± 0.31). As another important lymphangiogenetic factor, the average histoscore of podoplanin distribution was 7.34 ± 0.87 in all ESCC samples in present study, and their histoscores were also higher (10.08 ± 1.28) in NF-κB-high patients and lower (5.49 ± 1.05) in NF-κB-low patients (*p *= 0.008). Thus, LVD was significantly positively associated with NF-κB expression, but negatively associated with Notch1 expression. Consistent with this, VEGF-C expression was positively correlated with NF-κB and negatively correlated with Notch1 (Figure 3). To directly link NF-κB and Notch1 expression with lymphangiogenesis in ESCC, we performed a multiple factors analysis of LVD. As shown in Table 3, differences in LVD status were significantly correlated with expression of NF-κB, Notch1 and VEGF-C, independent of T stage, sex, age, and differentiation status of tumor cells. Moreover, a multiple factors analysis of VEGF-C, which is a key factor in tumor-induced lymphangiogenesis, revealed a positive association of VEGF-C status in ESCC tissue with the expression of NF-κB and a negative association with the expression of Notch1, independent of T stage, sex, age, and tumor cell differentiation status (Table 4).

**Table 3:** Multivariate analysis of LVD (VEGF-R3) in ESCC (logistic regression model)

Variable	*β*	HR (95% CI)	*P*
NF-κB	1.659	5.255 (1.296-21.300)	0.020
Notch1	-0.607	0.545 (0.329-0.904)	0.019
VEGF-C	0.583	1.791 (1.021-3.144)	0.042
T stage	-0.353	0.793 (0.442-1.118)	0.136
Sex	-1.548	0.213 (0.035-1.285)	0.092
Age	0.411	1.509 (0.092-24.751)	0.773
Differentiation	1.659	0.509 (0.099-2.627)	0.420

Abbreviations: HR, hazard ratio; CI, confidence interval of the estimated HR.

**Table 4:** Multivariate analysis of VEGF-C in ESCC (logistic regression model)

Variable	*β*	HR (95% CI)	*P*
NF-κB	1.930	6.889 (1.269-37.394)	0.025
Notch1	-0.605	0.546 (0.331-0.902)	0.018
T stage	0.765	2.149 (0.593-7.783)	0.244
Sex	0.371	1.450 (0.846-2.484)	0.176
Age	0.026	1.026 (0.969-1.088)	0.376
Differentiation	0.511	1.667 (0.607-4.580)	0.321

Abbreviations: HR, hazard ratio; CI, confidence interval of the estimated HR.

### Association of NF-κB expression with Notch1 expression in ESCC

**Figure 4:**
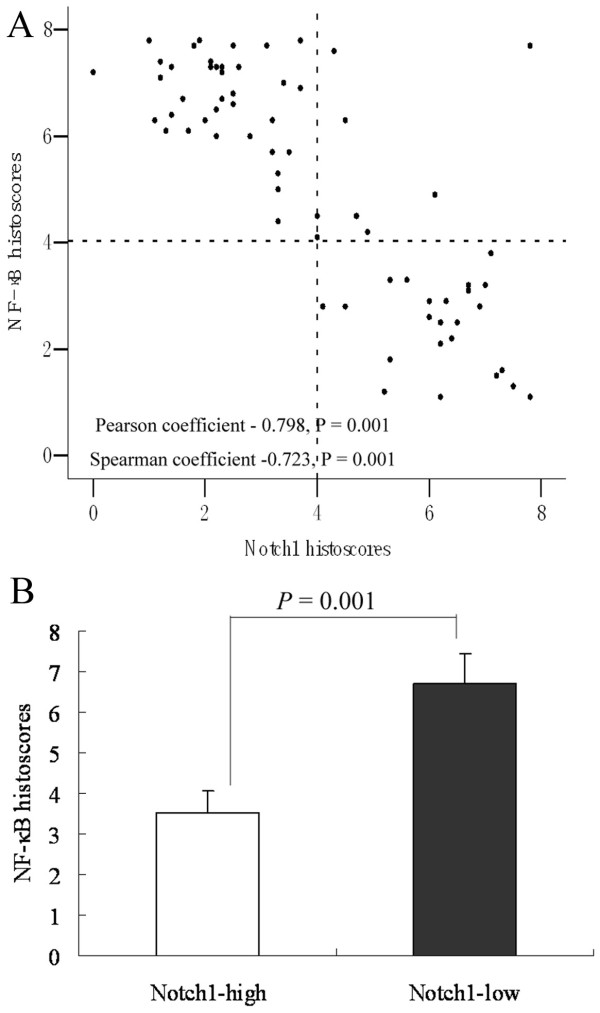
**Association of NF-κB expression with Notch1 expression in ESCC**. (A) NF-κB expression was negatively correlated with Notch1 expression in ESCC tissue. (B) The mean histoscore of NF-κB expression was lower in ESCC tissue with high levels of Notch1 expression (3.52 ± 0.53) than in those with low levels of Notch1 expression (6.71 ± 0.74; *P *< 0.05).

Collectively, our data suggested a significant correlation between NF-κB and Notch1 expression in ESCC tissues (Pearson coefficient, 0.798; *P *= 0.001; Spearman coefficient, -0.723; *P *= 0.001; Figure 4A). Lower NF-κB histoscores were observed in Notch1-high patients (3.52 ± 0.53), whereas higher NF-κB histoscores were found in Notch1-low patients (6.71 ± 0.74; Figure 4B). These results indicate that up-regulation of NF-κB is associated with down-regulation of Notch1 in ESCC.

## Discussion

Esophageal cancer is a disease with poor prognosis. Of the many prognostic factors identified to date, lymph node metastasis is one of the most significant, and tumor-associated lymphangiogenesis is believed to be a crucial prognostic factor for patient outcome [19,20]. VEGF-C has been characterized as a lymphangiogenic growth factor and has been shown to signal through the receptor, VEGFR-3 [21]. Moreover, there is a positive relationship between the expression of VEGF-C and the prognosis of patients with ESCC [20]. However, the precise mechanisms that underlie the development of tumor-associated lymphangiogenesis in ESCC are far from clear.

Recent accumulating evidence suggests that the NF-κB signaling pathway plays a critical role in carcinogenesis, protection from apoptosis, and chemoresistance in a number of cancer types, including head and neck cancer, breast cancer, and esophageal carcinoma [22-24]. NF-κB, which is retained in the cytoplasm through association with IκBα, is liberated upon phosphorylation of IκBα, whereupon it enters the nucleus to regulate the expression of genes involved in cell apoptosis and proliferation [25]. Importantly, NF-κB appears to be one of the main molecular mechanisms responsible for tumor formation and progression [26]. NF-κB is reported to be associated with invasive angiogenesis in cancer [27], and lymphatic endothelial cells express a set of specific markers (e.g., VEGF-C and VEGFR-3) [28]. On the basis of these observations, we assessed the relationships between intratumoral NF-κB and VEGFR-3 or VEGF-C expression in ESCC, in an effort to demonstrate the association of NF-κB with tumor-induced lymphangiogenesis. Our demonstration of a positive link between high levels of NF-κB expression and LVD and VEGF-C suggests that NF-κB may contribute to tumor-associated lymphangiogenesis in ESCC. The mechanistic aspect of the linkage between NF-κB and LVD was supported by the report that activation of NF-κB followed by sequential up-regulation of VEGFR-3 expression in cultured lymphatic endothelial cells and increasing of proliferation and migration, it suggested that induction of NF-κB enhanced the responsiveness of preexisting lymphatic endothelium to VEGFR-3 binding factors and resulted in lymphangiogenesis [29]. Interestingly, LVD reduced prominently in lungs of mice lacking p50 subunit of NF-κB, which demonstrated the important role of p50 subunit of NF-κB in regulating the expression of VEGFR-3 [30]. Regarding to the above molecular changing were found in inflammation-induced lymphangiogenesis, further research will be required to confirm the mechanistic aspect between NF-κB and LVD in tumor-associated lymphangiogenesis.

In contrast, we found that the expression of Notch1, which is involved in regulating vascular development, was negatively correlated with the lymphatic markers, VEGFR-3 and VEGF-C. These findings seemingly contradict those of a previous study, which reported that Notch signaling is positively correlated with VEGFR-3 and other lymphatic endothelial cell markers in physiological lymphangiogenesis [31]. The role of Notch1 in various tumors has been obscure, although researchers have suggested that Notch1 might contribute to guiding endothelial cells through the cell fate decisions needed to form and maintain a functional vascular network [32]; consistent with such a role, multiple connections between the VEGF system and the Notch signaling cascade have been previously described [33]. In a malignant environment, such as invasive breast carcinoma, cleaved (activated) Notch1 has been observed in a subset of lymphatic endothelial nuclei, indicating that Notch1 is not only expressed but is activated in tumor lymphatic vessels [31]. However, how Notch signaling participates in pathological tumor lymphangiogenesis remains unclear. Our finding that Notch1 expression is negatively associated with high expression of VEGF-C and VEGFR-3 in ESCC may indicate that down-regulation of Notch1 signaling contributes to tumor-induced lymphangiogenesis.

## Conclusions

Our findings demonstrate that high NF-κB and low Notch1 expression are correlated with high expression of VEGFR-3 (a marker of LVD) and VEGF-C, in ESCC patients, revealing an inverse relationship between Notch1 and NF-κB signaling and tumor-induced lymphangiogenesis. Taken together, our findings imply that Notch1 and NF-κB signaling have counter-acting roles in tumor-induced lymphangiogenesis in ESCC, and suggest that Notch may differentially regulate physiological and tumor-induced lymphangiogenesis.

## List of abbreviations

VEGF: vascular endothelial growth factor; VEGF-C: vascular endothelial growth factor C; ESCC: esophageal squamous cell cancer; VEGFR-3: Vascular endothelial growth factor receptor 3.

## Competing interests

The authors declare that they have no competing interests.

## Authors' contributions

The authors contributed to this study as follows: CS, ZC and HL conceived of the study; CS, YS, YL, YL and BZ performed experiments; ZC and LC analyzed data and prepared the figures; CS, ZC and HL drafted the manuscript. All authors have read and approved the final manuscript.
